# Supplementary material for: Plasticity in Intrinsic Excitability of Hypothalamic Magnocellular Neurosecretory Neurons in Late-Pregnant and Lactating Rats
Source: Int J Mol Sci. 2021 Jul 1;22(13):7140. doi: 10.3390/ijms22137140 (PMC8268815; doi:10.3390/ijms22137140)
Supplement: Supplementary file 1 [file ijms-22-07140-s001.zip › ijms-1265305-supplementary.pdf]

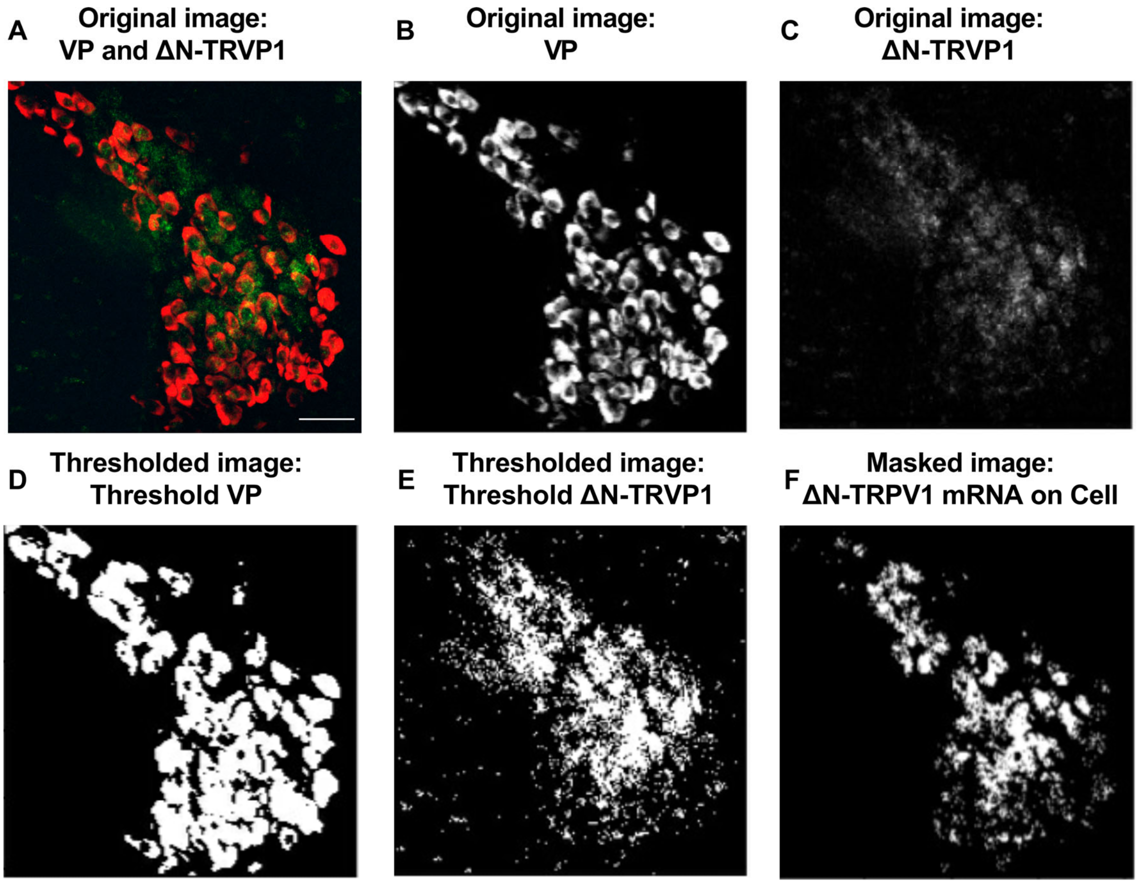

Supplementary Figure S1. Example of the analysis of vasopressin mRNA and ΔN-TRPV1 mRNA expression in the supraoptic nucleus of non-pregnant and late-pregnant rats. A. Example micrograph of vasopressin mRNA and ΔN-TRPV1 mRNA labelling in the supraoptic nucleus as shown in Figure 8B, generated by compression of Z-stacks using ImageJ Fiji Z-projection Max intensity. **B and C.** Images generated by splitting the image in panel A into the vasopressin mRNA channel (B) and ΔN-TRPV1 mRNA channel (C). **D and E.** Thresholded images for vasopressin mRNA expression from panel B and ΔN-TRPV1 mRNA from panel C, generated using Cell Profiler™. **F.** Masked image created by overlaying the two thresholded images in panels D and E, showing only pixels that were co-labelled for vasopressin mRNA and ΔN-TRPV1 mRNA. Note that vasopressin mRNA labelling did not allow differentiation between the borders of individual cells in all sections. Therefore, it was not possible to quantify of numbers of ΔN-TRPV1 mRNA-positive vasopressin neurons.

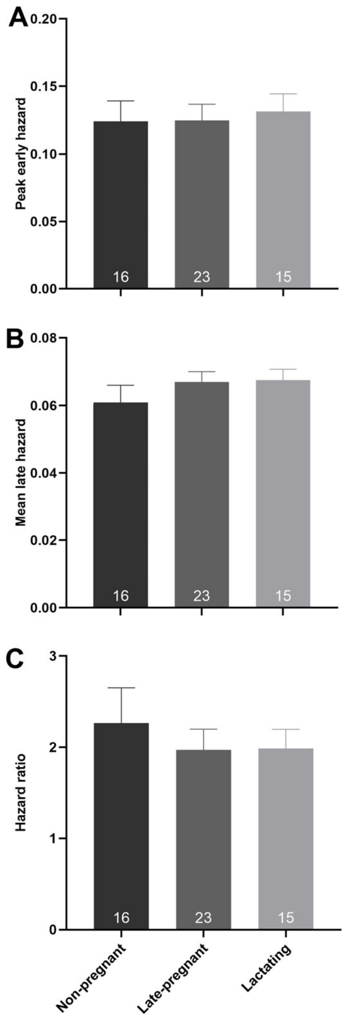

Supplementary Figure S2. A – C. Mean (± SEM) peak early hazard (A), mean late hazard (B) and peak early:mean late hazard ratio (C) of phasic vasopressin neurons in non-pregnant, late-pregnant and lactating rats. There was no effect of reproductive status on the peak early hazard (H = 0.17, P = 0.92, one-way ANOVA on ranks), mean late hazard (H = 4.27, P = 0.12) or hazard ratio (H = 0.41, P = 0.82) of phasic vasopressin neurons in non-pregnant, late-pregnant and lactating rats.

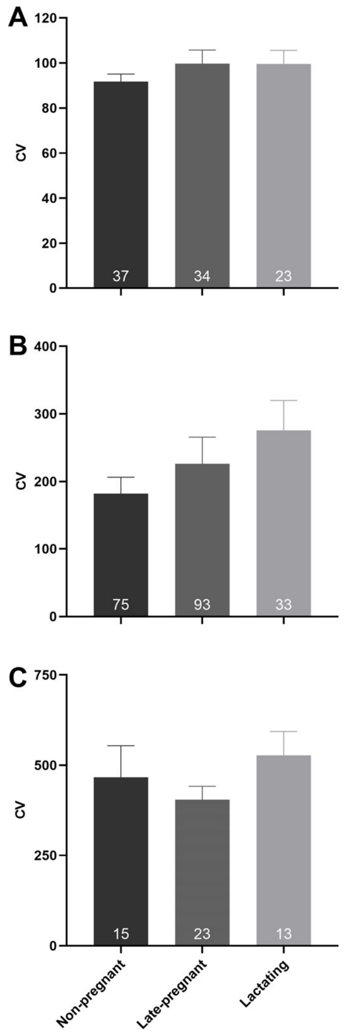

Supplementary Figure S3. A – C. Mean (± SEM) coefficient of variation of inter-spike interval of oxytocin neurons (A), vasopressin neurons (B) and phasic vasopressin (C) neurons. There was no effect of reproductive status on the CV of oxytocin neurons ($H = 0.76$, $P = 0.69$, one-way ANOVA on ranks), vasopressin neurons ($H = 5.43$, $P = 0.07$), or phasic vasopressin neurons ($H = 2.73$, $P = 0.25$).
